# Titanium dioxide nanotubes applied to conventional glass ionomer cement influence the expression of immunoinflammatory markers: An in vitro study

**DOI:** 10.1016/j.heliyon.2024.e30834

**Published:** 2024-05-08

**Authors:** João Pedro Rangel-Coelho, Pedro Viel Gogolla, Maria Davoli Meyer, Lucas Carvalho Simão, Bruna Carolina Costa, Renato Côrrea Viana Casarin, Mauro Pedrine Santamaria, Lucas Novaes Teixeira, Daiane Cristina Peruzzo, Paulo Noronha Lisboa-Filho, Francisco Humberto Nociti-Jr, Kamila Rosamilia Kantovitz

**Affiliations:** a Faculdade São Leopoldo Mandic (SLMANDIC), Rua José Rocha Junqueira 13, Swift, Campinas, SP, 13045-755, Brazil; b Department of Physics, School of Science, State University Júlio de Mesquita (UNESP), Av. Engenheiro Luís Edmundo Carrijo Coube 2085, Bauru, SP, 17033-360, Brazil; c Department of Prosthodontics and Periodontics, Division of Periodontics, Piracicaba Dental School, State University of Campinas (FOP-UNICAMP), Av. Limeira 901, Areião, Piracicaba, SP, 13414-903, Brazil; d College of Dentistry, University of Kentucky, 1095 Veterans Dr, Lexington, KY, USA; e American Dental Association Science and Research Institute - ADASRI, Cellular and Molecular Biology Research Group, Innovation and Technology Research, 100 Bureau Dr, Gaithersburg, MD, 20899, USA

**Keywords:** Glass ionomer cements, Titanium dioxide, Nanotechnology, Fibroblasts, Proteome, Interleukins

## Abstract

**Objectives:**

To assess the impact of different concentrations TiO_2_-nt incorporated into a glass ionomer cement on the proliferation, mitochondrial metabolism, morphology, and pro- and anti-inflammatory cytokine production of cultured fibroblasts (NIH/3T3), whether or not stimulated by lipopolysaccharides (LPS-2 μg/mL, 24 h).

**Methods:**

TiO_2_-nt was added to KM (Ketac Molar EasyMix™, 3 %, 5 %, 7 % in weight); unblended KM was used as the control. The analyses included: Cell proliferation assay (n = 6; 24/48/72h); Mitochondrial metabolism assay (n = 6; 24/48/72h); Confocal laser microscopy (n = 3; 24/48/72h); Determination of biomarkers (IL-1β/IL-6/IL-10/VEGF/TNF) by using both multiplex technology (n = 6; 12/18 h) and the quantitative real-time PCR assay (q-PCR) (n = 3, 24/72/120 h). The data underwent analysis using both the Shapiro-Wilk and Levene tests, and by generalized linear models (α = 0.05).

**Results:**

It demonstrated that cell proliferation increased over time, regardless of the presence of TiO_2_-nt or LPS, and displayed a significant increase at 72 h; mitochondrial metabolism increased (p < 0.05), irrespective of exposure to LPS (p = 0.937); no cell morphology changes were observed; TiO_2_-nt reverted the impact of KM on the secreted levels of the evaluated proteins and the gene expressions in the presence of LPS (p < 0.0001).

**Conclusions:**

TiO_2_-nt did not adversely affect the biological behavior of fibroblastic cells cultured on GIC discs.

## 1 Introduction

Glass ionomer cements (GICs) are categorized as acid-base cements materials, comprising basically of fluoride-aluminum-silicate powder and polyacrylic acid liquid [1]. Their favorable chemical, physical and biological properties have made them widely used in the dental clinic for the cementation of indirect restorations and orthodontic brackets, as a liner, sealant, for minimally invasive restorative treatments [1,2], nevertheless survival rates are about 50 % of cases over 3 years [3]. This considerable flaw is related to their high sensitivity to moisture during the initial 24 h of the setting process, and consequent hydrolytic degradation, which may compromise their mechanical properties and color stability [2,4].

In this context, the advent of nanotechnology stands out as a pivotal advancement in both medicine and dentistry [5,6]. Smaller particle sizes (less than 100 nm in diameter) have provided greater surface area and improved the interaction among molecules [7]. TiO_2_ nanoparticles—an inorganic particle in the anatase form—have been proposed as a key adjunct to ensure favorable physicomechanical properties of dental materials [[8], [9], [10], [11], [12], [13], [14], [15]]. Their addition to GIC has also been shown to affect bacterial growth [8,16,17]. For instance, in vitro studies have reported that TiO_2_-nt significantly improved the compressive strength, surface microhardness, and solubility resistance of GIC, as well as its fluoride release levels, color opacity, and radiopacity [11,13,15,18], and reduced weight loss after wear [13]. On the other hand, TiO_2_-nt did not affect the adhesion properties of GICs to dentin, the aluminum release levels, or the morphology of fibroblasts [11,[13], [14], [15]]. Overall, greater knowledge has been gained ongoingly in this area to improve GIC performance [4,[8], [9], [10], [11], [12], [13], [14], [15], [16], [17], [18]].

Furthermore, dental restoration procedures must take biological principles into account; hence, one should assess biological impact of a new dental material and in vitro models offer established assays to address these questions. As a restorative material, GIC might eventually be in direct contact with the gingival tissues, which is populated by fibroblasts that will modulate tissue repair and inflammatory response by producing bioactive factors, including interleukin (IL)-1β, 10, and 6, and tumoral necrosis factor alpha (TNF-α) [[19], [20], [21]]. These biomarkers feature interconnected function and are involved in several biological processes [[22], [23], [24], [25]].

Since lipopolysaccharides (LPS) have been implicated with the progression of inflammation, special attention has been given to LPS as an important modulator of the inflammatory response [[25], [26], [27], [28], [29], [30]]. LPS have been reported to trigger intracellular signaling responses that will ultimately lead to the secretion of inflammatory active agents and growth factors intended to control microbial infection [25,27,29]. Accordingly, efforts have been made to distinguish physiological expression of these mediators versus a response to the presence of dental materials [20,[31], [32], [33], [34], [35]]. Interestingly, nanostructures have been demonstrated to modulate cell responses [11,30,34,[36], [37], [38], [39], [40], [41], [42]]. However, scant knowledge is available on the influence of GICs containing TiO_2_-nt about biomarkers expression by fibroblasts (NIH/3T3) stimulated with LPS. Thus, the current investigation defined the effect of 3 %, 5 %, or 7 % TiO_2_-nt incorporated into a conventional high-viscosity GIC on fibroblasts cultures challenged with bacteria LPS.

## 2 Materials and methods

### 2.1 Sampling

Preliminary experiments defined sample sizes (n) for the study, while P value (5 %) and statistical power (80 %) were set by the G*Power software 3.1.9.7. One calibrated and experienced examiner performed the analysis under IRB approval (protocol #2022-0899).

### 2.2 Experimental design

The following groups were set: KM (Ketac Molar EasyMix™ by 3 M, batches #7294769 and #7294569) = control (unblended powder); KM + LPS = control (unblended powder) + LPS; KM+3%TiO_2_-nt; KM+3%TiO_2_-nt + LPS; KM+5%TiO_2_-nt; KM+5%TiO_2_-nt + LPS; KM+7%TiO_2_-nt; KM+7%TiO_2_-nt + LPS.

**Fig. 1:**
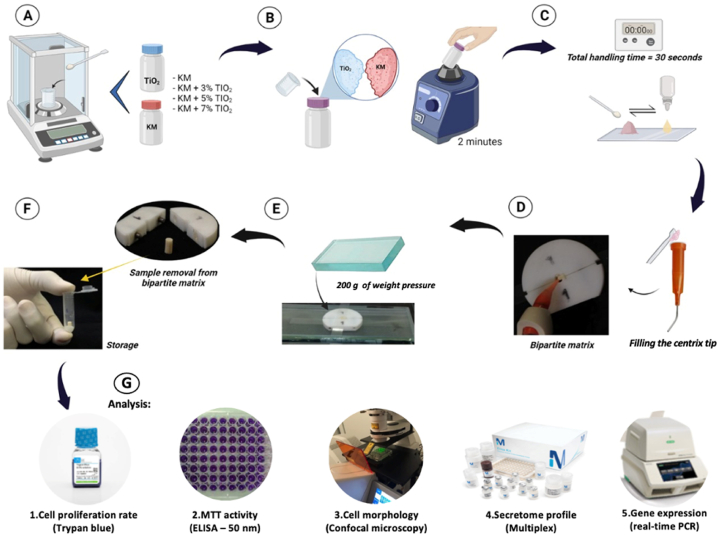
Methodology illustration: (a) determining GIC and TiO_2_-nt proportions on an accurate scale; (b) incorporating TiO_2_-nt into GIC; (c) GIC preparation (1:1 ratio); (d) GIC into molds with the assistance of a syringe (e) setting time – 6 min under a 200 g static load; (f) the samples were detached from the mold, coated with petroleum jelly for 24 h at 37 °C; (g) the analysis included: (1) cell proliferation rate (trypan blue; n = 6; at 24/48/72 h); (2) mitochondrial activity rate (MTT; n = 6; at 24/48/72 h); (3) cell morphology (Confocal microscopy) (n = 3; at 24/48/72 h); (4) secretome profile (Multiplex) (n = 6; at 12/18 h); and (5) gene expression (real-time PCR) (n = 6; at 24/72/120 h). Figure adapted from Kantovitz et al., 2023, and created using BioRender.com.

Fibroblast cells (NIH/3T3 cells) were cultured on pre-made discs containing or not TiO_2_-nt, and with or without stimulation by *Bacteroides forsythus* LPS. The following parameters were evaluated: (1) cell proliferation rate (trypan blue; n = 6; at 24/48/72 h); (2) mitochondrial activity rate (MTT; n = 6; at 24/48/72h); (3) cell morphology (confocal microscopy) (n = 3; at 24/48/72h); (4) secretome profile (Multiplex) (n = 6; at 12/18 h); and (5) gene expression (real-time PCR) (n = 6; at 24/72/120 h) (Fig. 1). GIC samples were subjected to ultraviolet light exposure for a duration of 15 min/surface for decontamination [11]. Experiments were performed in compliance with ISO 10993-5 (2009) recommendations [43]. A negative control group with cells plated without the GIC disc was used at all the experimental time points. LPS concentration used in this study was established through a preliminary dose-response proliferation assay using three different LPS concentrations (0.2, 2, and 20 μg/mL). The selected LPS concentration was 2 μg/mL at 24 h.

### 2.3 Incorporation of TiO_2_-nt into GIG powder

Nanotubes (≈20 nm in size - 10 nm diameter) were created [44], after which their structural and morphological characteristics were characterized [15]. A highly precise scale was used to define the mixtures (Fig. 1). Subsequently, nanotubes were manually incorporated into the KM powder and thoroughly mixed with the assistance of a vortex device (Biomixer, Tafl, CA, USA) for 2 min, following a method described previously [11,[13], [14], [15],^17^].

### 2.4 Specimens

KM components were prepared as described elsewhere [15] to determine and validate the quantity of materials used: KM(0.147 ± 0.003g); KM+3%TiO_2_-nt(0.167 ± 0.005g); KM+5%TiO_2_-nt(0.172 ± 0.004g); KM+7%TiO_2_-nt(0.168 ± 0.003g); and volume of liquid dispensed (0.085 ± 0.005g). Next, 1:1 ratio was used and homogenized as recommended by the manufacturer [1], and the samples prepared as described elsewhere [15] (Fig. 1). A thin layer of petroleum jelly coated the samples before they were stored for 24 h [1,15].

### 2.5 Cell culture

Murine fibroblasts (NIH/3T3) from the 13th and 14th passages were used in the experiments and cultured as described elsewhere [11]. Twenty-four hours post-plating, the culture medium was exchanged to 5 % FBS, along with antibiotics and data collected at this time point with or without LPS, using a challenge assay.

### 2.6 Cell proliferation assay (trypan blue; n = 6/group)

GIC samples with or without TiO_2_-nt (3 %, 5 % and 7 %) were individually placed in 48-well plates (Corning Costar, # CLS3549). Cells were plated in triplicate (1 × 10^3^ cells/well) and cultured for 24 h. The growth medium was switched to 5 % FBS and antibiotics for data collection. Total number of cells (viable and non-viable) was determined with a hemocytometer and trypan blue (24/48/72 h). Concisely, the cells were trypsinized, centrifuged, and resuspended in phosphate buffer saline (PBS). The viable and non-viable cell numbers were obtained after an incubation period of 3–5 min in the presence of trypan blue [45].

### 2.7 Mitochondrial activity rate (MTT; n = 6/group)

After the materials were cured, the samples were positioned individually into 48-well culture plates and the cells plated (1.5 × 10^4^ cells/well) as described before [11]. Data was collected (24/48/72 h) as recommended by the manufacturer and described elsewhere [46].

### 2.8 Cell morphology (confocal microscopy) (n = 3/group)

Fibroblasts (NIH/3T3) were cultured in 48-well plates in duplicate on GIC discs with or without TiO_2_-nt (3 %, 5 %, and 7 %). Cells were maintained for 24 h as previously reported [11]. Next, the culture medium was and samples imaged (24/48/72 h) by confocal microscopy as described elsewhere [47].

### 2.9 Multiplex secretome analysis (n = 3/group)

NIH/3T3 were seeded (1 × 10^3^ cells/well) and cultured on GIC discs with or without nanotubes for 24 h. Culture media without supplementation was collected after 12 and 18 h, after which it was stored at −80 °C for later investigation of biomarker expression using the MAGPIX system (Milliplex, Denton, TX, USA). Levels of IL-1β/6/10, VEGF, TNF were determined using a previously described protocol [46].

### 2.10 Transcript levels (n = 3/group)

NIH/3T3 were seeded (1 × 10^3^ cells/well in 24-well plates) and cultured on GIC discs with or without nanotubes and LPS 24 h. Transcript levels of IL-1β/6/10, VEGF, TNF-α were defined as previously described [47]. Table 1 presents the primer sequences.

**Table 1:** Primers.

*Targets*	Sequence 5′-3′
16SRNA	CGGCAAGCTAATCTCTGAAA**/**GCCCCTAAAAGG TTACCTCA
IL-1β	TGTGCAATGGCAATTCTGAT**/**GGTACTCCAGAAGACCAGAGGA
IL-6	GTCCTTCAGAGAGATACAGAAACT**/**AGCTTATCTGTTAGGAGAGCATTG
IL-10	AAGGCAGTGGAGCAGGTGAA**/**CCAGCAGACTCAATACACAC
VEGF	TGCAGATTATGCGGATCAAACC**/**TGCATTCACATTTGTTGTGCTGTAG
TNF-α	TGCCTATGTCTCAGCCTCTTC**/**GGTCTGGGCCATAGAACTGA

### 2.11 Statistics

Data normal distribution was defined by appropriate tests (Shapiro-Wilk & Levene's) (p ≤ 0.05). Assessments of cell proliferation, mitochondrial metabolism (MTT), and biomarkers level were done by generalized linear models to ascertain the impact of time, LPS and TiO_2_-nt on fibroblast cells (α = 0.05). Qualitative analyses were performed for morphological data (confocal microscopy). Statistics assessments used the R software.

## 4 Results

### 4.1 Cell proliferation assay (trypan blue)

Data analysis showed that time significantly affected cell numbers, regardless of the presence of GIC or LPS (p < 0.05), pointing out that the highest cell numbers were observed at 72 h. Furthermore, at 24 h, our findings show that LPS lead to a significantly rise in cell numbers in the GIC (only) and negative control (no disc) (p < 0.05). On the other hand, differences were not significant for the groups containing TiO_2_-nt and LPS (p > 0.05). Additionally, data revealed that LPS treatment did not significantly change cell numbers across the groups at 48 or 72 h, except for the negative control (no disc) at 48 h, which a higher number of cells was also observed in the presence of LPS (p < 0.05). Further data analysis indicated that the groups containing TiO_2_-nt regularly exhibited no discernible differences when treated with or without LPS, implying that the presence of TiO_2_-nt did not influence the proliferative rates of NIH/3T3 cells cultured on GIC discs with or without nanotechnology. Table 2 summarizes the findings of the proliferative rates across the experimental groups.

**Table 2:** Mean (cell number x10^4^) and standard deviation (SD) values for NIH/3T3 fibroblast proliferative rates on experimental discs with and without LPS at 24, 48 and 72 h (n = 6/group).

Time	**Experimental groups**	Cell proliferation	
		With LPS	Without LPS
24 h	Cells (negative control)	0.47 (0.04) **Aa**	0.33 (0.04) **Bab**
	KM	0.36 (0.06) **Ab**	0.29 (0.04) **Bbc**
	KM+3%TiO_2_-nt	0.27 (0.04) **Ac**	0.25 (0.03) **Ac**
	KM+5%TiO_2_-nt	0.36 (0.05) **Ab**	0.34 (0.03) **Aa**
	KM+7%TiO_2_-nt	0.30 (0.04) **Ac**	0.29 (0.04) **Abc**
48 h	Cells (negative control)	a0.76 (0.06) **Aa**	a0.63 (0.04) **Bab**
	KM	a0.56 (0.04) **Ac**	a0.60 (0.05) **Ab**
	KM+3%TiO_2_-nt	a0.57 (0.05) **Ac**	a0.67 (0.04) **Aab**
	KM+5%TiO_2_-nt	a0.61 (0.05) **Abc**	a0.58 (0.23) **Ab**
	KM+7%TiO_2_-nt	a0.67 (0.05) **Aab**	a0.71 (0.05) **Aa**
72 h	Cells (negative control)	ab1.46 (0.05) **Aa**	ab1.39 (0.07) **Aa**
	KM	ab1.40 (0.06) **Aa**	ab1.34 (0.06) **Aa**
	KM+3%TiO_2_-nt	ab1.29 (0.04) **Aa**	ab1.45 (0.03) **Aa**
	KM+5%TiO_2_-nt	ab1.41 (0.04) **Aa**	ab1.44 (0.07) **Aa**
	KM+7%TiO_2_-nt	ab1.39 (0.06) **Aa**	ab1.37 (0.05) **Aa**

Different uppercase letters indicate statistically significant differences horizontally (among LPS categories), and different lowercase letters indicate statistically significant differences vertically (among TiO2-nt concentrations), within each time category (p ≤ 0.05). p(TiO2-nt)<0.0001; p(LPS) = 0.0025; p(time) < 0.0001; p(TiO2-nt vs. LSP) = 0.0038; p(TiO2-nt vs. time) < 0.0001; p(LSP vs. time) = 0.0011; p(TiO2-nt vs. LSP vs. time) = 0.0801. KM = positive control.

a Significant difference at 24 h, considering the same TiO2-nt and LPS conditions (p ≤ 0.05).

b Significant difference at 48 h, considering the same TiO2-nt and LPS conditions (p ≤ 0.05).

### 4.2 Mitochondrial metabolism assay (MTT)

Table 3 shows the data analysis for cellular metabolism. There was a significant interaction between GIC with TiO_2_-nt and time (p = 0.0327). Study data showed that LPS did not impact the metabolic activity of NIH/3T3 cells, irrespective of TiO_2_-nt (p = 0.2912). The results of the TiO_2_-nt concentration comparisons showed that metabolic activity depended only on time, and the highest metabolic activity was observed at 72 h (p < 0.001). Overall, our findings demonstrated that TiO_2_-nt did not alter MTT of NIH/3T3 cells, compared with negative control (only cells) and the GIC groups.

**Table 3:** Mean (cell number x10^4^) and standard deviation (SD) values for NIH/3T3 fibroblast metabolic activity (MTT) on experimental discs with and without LPS at 24, 48, 72 h (n = 6/group).

Time	Experimental groups	Metabolic Activity (MTT)		
		With LPS	Without LPS	
24 h	Cells (negative control)	0.56 (0.01) **A**	0.60 (0.04)	**a**
	KM	0.48 (0.12) **A**	0.52 (0.09)	**ab**
	KM+3%TiO_2_-nt	0.47 (0.08) **A**	0.44 (0.06)	**b**
	KM+5%TiO_2_-nt	0.61 (0.08) **A**	0.51 (0.04)	**a**
	KM+7%TiO_2_-nt	0.44 (0.02) **A**	0.45 (0.17)	**b**
48 h	Cells (negative control)	a1.51 (0.12) **A**	a1.52 (0.22)	**a**
	KM	a1.16 (0.11) **A**	a1.44 (0.06)	**ab**
	KM+3%TiO_2_-nt	a1.20 (0.15) **A**	a1.20 (0.24)	**b**
	KM+5%TiO_2_-nt	a1.34 (0.09) **A**	a1.37 (0.35)	**ab**
	KM+7%TiO_2_-nt	1.20 (0.11) **A**	1.20 (0.15)	**b**
72 h	Cells (negative control)	ab1.84 (0.14) **A**	ab2.01 (0.09)	**a**
	KM	ab1.91 (0.37) **A**	ab1.67 (0.57)	**ab**
	KM+3%TiO_2_-nt	ab1.79 (0.49) **A**	ab2.04 (0.20)	**a**
	KM+5%TiO_2_-nt	ab1.83 (0.54) **A**	ab1.41 (0.42)	**b**
	KM+7%TiO_2_-nt	ab1.47 (0.24) **A**	ab1.89 (0.38)	**ab**

Different uppercase letters indicate statistically significant differences horizontally (among LPS categories), and different lowercase letters indicate statistically significant differences vertically (among TiO2-nt concentrations), within each time category (p ≤ 0.05). There was a significant interaction between GIC with TiO2-nt and time (p = 0.0327). p(TiO2-nt) = 0.0008; p(LPS) = 0.9367; p(time) < 0.0001; p(TiO2-nt vs. LSP) = 0.2912; p(LSP vs. time) = 0.7562. An interaction among factors was observed: p(TiO2-nt vs. time) = 0.0327; KM = positive control.

a Significant difference at 24 h, considering the same TiO2-nt and LPS conditions (p ≤ 0.05).

b Significant difference at 48 h, considering the same TiO2-nt and LPS conditions (p ≤ 0.05).

### 4.3 Cellular adhesion/morphology by confocal microscopy analysis

**Fig. 2:**
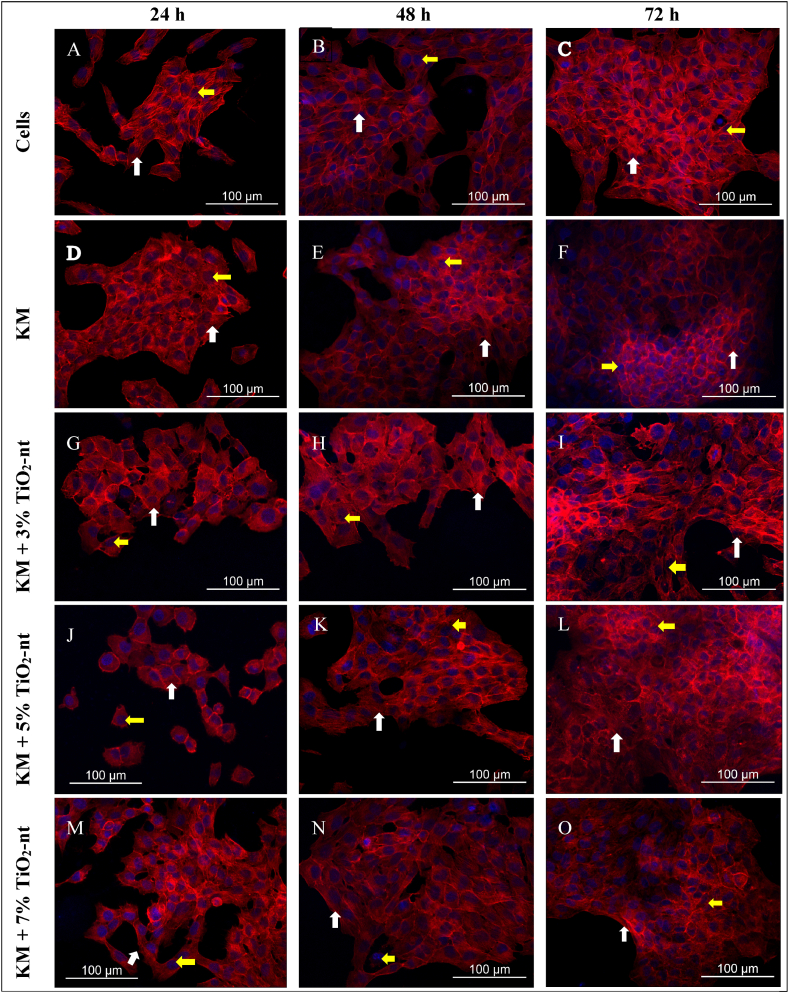
Confocal microscopy qualitative analysis of the morphology of fibroblast cells cultured on GIC discs with or without 3 %, 5 %, and 7 % in weight of TiO_2_-nt not exposed to LPS, at 24 (A, D, G, J, M), 48 (B, E, H, K, N), and 72 h (C, F, I, L). Actin cytoskeleton is stained red (phalloidin conjugated to Alexa 555) (white arrows), whereas cell nuclei are blue (DAPI (yellow arrows).

**Fig. 3:**
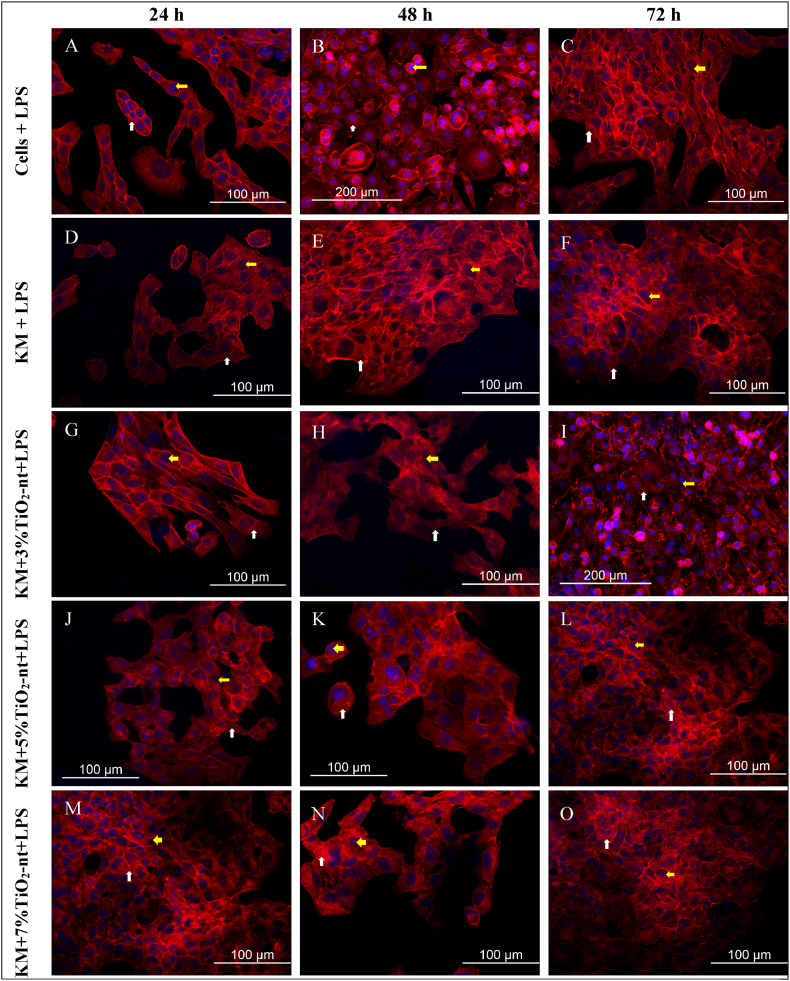
Confocal microscopy qualitative analysis of the morphology of fibroblast cells cultured on GIC discs with or without 3 %, 5 %, and 7 % in weight of TiO_2_-nt exposed to LPS, at 24 (A, D, G, J, M), 48 (B, E, H, K, N), and 72 h (C, F, I, L). Actin cytoskeleton is stained red (phalloidin conjugated to Alexa 555) (white arrows), whereas cell nuclei are blue (DAPI (yellow arrows).

NIH/3T3 cells were able to adhere to the substrate in all the experimental groups, regardless of the presence of either GIC or TiO_2_-nt, and irrespective of treatment with LPS. Cell adhesion was more evident at 48 and 72 h. In addition, it was noted that there were no evident distinctions regarding cell morphology across the experimental groups. Fig. 2 (A-O) and Fig. 3 (A-O) illustrate representative images from the confocal microscopy analyses.

### 4.4 Secretome analysis

**Fig. 4:**
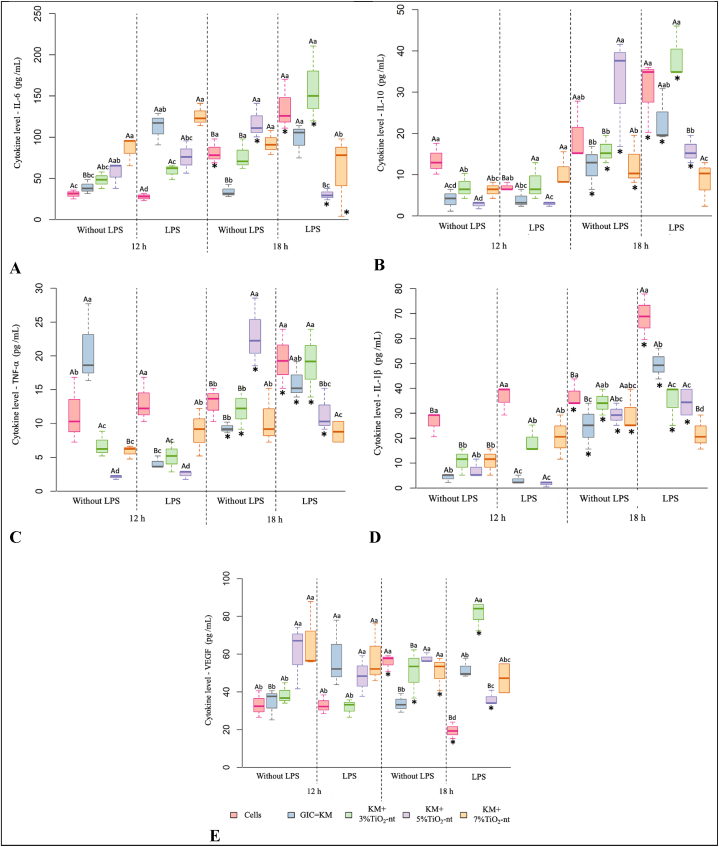
Box plot of secretome biomarker expression by multiplex assay (in pg/mL) and by fibroblast cells (NIH/3F3) according to TiO_2_-nt concentration, LPS challenging condition, and time (12 and 18 h). *^Significant difference at 12 h under the same TiO2−nt and LPS conditions (p ≤ 0.05). A triple interaction among factors was observed: p (TiO2−nt X LSP X time) < 0.0001. Different uppercase letters mean statistically significant differences among the LPS challenging conditions within each experimental group. Different lowercase letters mean statistically significant differences among TIO2−nt concentrations within each time category (p ≤ 0.05).^

The cytokine multiplex assay showed that the selected inflammatory markers were expressed and secreted by NIH/3T3 cells under the conditions of the study (Fig. 4A–E). Data analysis showed that IL-6 levels were significantly increased over time and by LPS treatment at 12 and 18 h (p < 0.05). There was a triple interaction among TiO_2_-nt, LPS, and the time factors (p = 0.0015). In addition, TiO_2_-nt significantly reduced the levels of IL-6 at 3 % and 5 % in the presence of LPS at 12 h, and at 5 % and 7 % TiO_2_-nt at 18 h (p = 0.0002). In the absence of LPS, TiO_2_-nt increased the secreted levels of IL-6 at 5 % and 7 %, and at 3 %, 5 % and 7 %, at 12 h or 18 h, respectively, versus GIC alone (p < 0.0001). A triple interaction among TiO_2_-nt, LSP, and the time factors was detected for IL-10 (p = 0.0214). The secreted levels of cytokine were unaltered by LPS at 12 h; however, IL-10 levels were increased by LPS at 18 h (p < 0.001). In the presence of LPS, TiO_2_-nt significantly increased IL-10 levels at 3 % and 7 % at 12 h, and at 3 % at 18 h (p < 0.001), compared with GIC alone. In contrast, TiO_2_-nt significantly decreased IL-10 levels at 7 % at 18 h. In the absence of LPS, a noteworthy distinction was identified for the 3 % and 5 % TiO_2_-nt groups at 12 and 18 h, respectively, compared with GIC alone (p < 0.001). As expected, IL-1β was significantly increased by LPS treatment and by time (p < 0.001). Compared with GIC alone, the addition of 3 % and 7 % TiO_2_-nt significantly increased the levels of IL-1β at 12 h, whereas the addition of 3 %, 5 %, and 7 % TiO_2_-nt significantly decreased the levels of IL-1β at 18 h (p < 0.001). In the current investigation, TNF-α levels were significantly reduced by TiO_2_-nt in the presence of LPS, at all the evaluated concentrations, whereas no significant effect was observed at 18 h, except for the 5 % TiO_2_-nt (p < 0.0001). The treatment with LPS led to a marked elevation the expression of VEGF by fibroblasts (p = 0.0107), whereas time did not influence VEGF secretion (p = 0.8172), VEGF decreased with 3 % TiO_2_-nt at 12 h. Conversely, 5 % TiO_2_-nt significantly elevated VEGF at 18 h in the presence of LPS (p < 0.0001). In the absence of LPS, TiO_2_-nt increased VEGF secretion by NIH/3T3 cells across all experimental groups, except for the 3 % TiO_2_-nt at 12 h (p < 0.0001).

### 4.5 Gene expression analysis

**Fig. 5:**
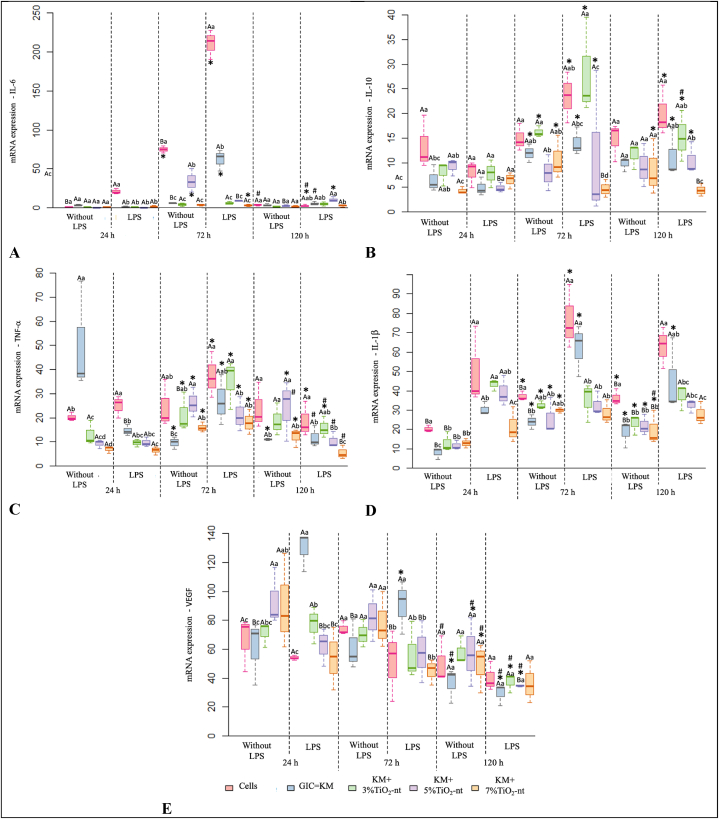
Box plot of the RT-qPCR validation of pro- and anti-inflammatory gene expression by fibroblast cells (NIH/3F3) according to TiO_2_-nt concentration, LPS-challenging condition, and time (24, 72, and 120 h). *^Significant difference at 24 h under the same TiO2−nt and LPS conditions (p ≤ 0.05). # Significant difference at 72 h under the same TiO2−nt and LPS conditions (p ≤ 0.05). Different uppercase letters mean statistically significant differences between the LPS challenging conditions, within each experimental group. Different lowercase letters mean statistically significant differences among the TiO2−nt concentrations, within each time category (p ≤ 0.05).^

Fig. 5 (A-E) reveled the cytokine expression of NIH/3T3 cells by the qPCR assay. Data analysis indicated a triple interaction among the TiO_2_-nt, LSP, and time factors for all the cytokines evaluated (p < 0.05). Differences were not significant among the groups for the mRNA levels of IL-6 in the presence of LPS, apart from the KM + 5 % TiO_2_-nt group (p < 0.0001). Regarding IL-10, there was no significant difference among the groups with and without LPS (p = 0.7028), except for the KM + 7 % TiO_2_-nt group, which showed a significantly lower gene expression of IL-10 in the presence of LPS at 72 and 120 h (p = 0.0377). In contrast, regarding IL-1β, there was a significant increase in gene expression in the presence of LPS for all the experimental groups at different the time points (p < 0.0374). Fig. 5D shows that GIC with no nanotechnology had lower TNF-α gene expression than other groups at 24 h. The same occurred with the KM containing 5 % and 7 % TiO_2_-nt at 120 h, demonstrating that nanotechnology did not alter the TNF-α gene expression pattern over time. As for VEGF, GIC alone promoted significantly higher gene expression levels by fibroblasts in LPS presence at 72 and 120 h, whereas GIC plus 5 % or 7 % TiO_2_-nt expressed significantly lower levels (p < 0.001) (Fig. 5E).

## 5 Discussion

*Fibroblast response to TiO*_*2*_*-nt in the presence of LPS:* Data analysis showed that TiO_2_-nt resulted in a greater proliferative and metabolic activity rate overtime. In addition, LPS significantly affected proliferation and metabolic activity of NIH/3T3 cells cultured on GIC discs, whereas the TiO_2_-nt-treated groups displayed a less evident of LPS. Consistently with the findings of the current study, it has been previously reported that TiO_2_-nt does not affect the biocompatibility of GIC in vitro [18,48,64]. In addition, KM + 5 % TiO_2_-nt was associated with the highest metabolic cell rate over time, facilitating the production of both collagenous and non-collagenous extracellular matrix [11]. In contrast, TiO_2_ nanoparticles have been reported to present cytotoxic properties [48,49]. The interaction mechanisms between nanostructures and living systems are still not fully understood. It has been suggested that the nanostructures penetrate cells through active or passive mechanisms and contribute to accelerating the host response to a foreign body. Characteristics that may affect how nanoparticles modulate the host response are: material type, size, format, surface and load type, coating, dispersal, agglomeration and concentration of nanostructures [37].

*Inflammatory markers are regulated by the presence of TiO*_*2*_*-nt:* It is recognized that nanomaterials safety and efficiency are directly related to its ability to modulate the inflammatory response. The current investigation discovered that GIC containing TiO_2_-nt modulated the expression of immune-inflammatory markers by NIH3/T3 in the presence of bacteria LPS (Fig. 4 A-E; Fig. 5A–E). In general, immune-inflammatory markers play key roles biological systems, including the regulation of the activity of white blood cells, modulation of T-lymphocytes function and apoptosis, production of chemokines against pathogens, modulation of the host response to trauma, recruitment of cells and promotion of angiogenesis [19,[50], [51], [52]]. Therefore, determining how TiO_2_-nt incorporated to GIC affect the expression of inflammatory markers by NIH/3T3 cells will provide valuable information to expand our understanding on the biological implications involved with such association. In general, our findings show that GIC alone affected the transcript levels of selected inflammatory markers, whereas TiO_2_-nt reverted this response. As expected, LPS significantly increased the levels of the evaluated cytokines at 12 and 18 h, with TiO_2_-nt reversing the effect of LPS on cultured on GIC discs for all markers at 12 h, except TNF-α. A similar effect was observed in the absence of LPS for the following markers: IL-6 (18 h), IL-10 (18 h), TNF-α (12 h), and IL-1β (18 h) (Fig. 4 A-E; Fig. 5A–E).

As a member of the IL-1 family [53], IL-1β was demonstrated to be a key player in the pathogenesis of several conditions, including osteoarthritis [54]. The biological effect of IL-1***β*** occurs based on its interaction with membrane receptors [55,56]. TNF-***α*** has also been reported to be a key inflammatory marker, and to bind to TNF-R1 and TNF-R2 that are expressed by almost every nucleated cell. IL-6 was defined as a pro-inflammatory mediator involved in a number of biological processes, such as bone metabolism, liver pathologies, tumors and intraocular neovascular disorders [25]. In contrast to IL1-β, TNF-α and IL-6, IL-10 has been described to have anti-inflammatory properties, playing a central role by limiting the immune-inflammatory response [57]. VEGF is a critical factor regulating physiological angiogenesis during numerous complex conditions and may serve as a target for prevention of angiogenesis, and visual loss in age-related macular degeneration [58].

Previous reports suggested the potential of TiO_2_-nt to alter the expression of biological markers involved in the regulation of the inflammatory processes by activating T- and B-lymphocytes in a dose-dependent way after 24 h [[27], [28], [29], [30],^58^]. Importantly, the authors found that cell activation leading to increased expression of biological markers lasted for up to 14 days. Furthermore, intratracheal administration of TiO_2_ nanoparticles led to increased levels of MIPs (macrophage inflammatory proteins) and MCPs (monocyte chemoattractant proteins) [59]. Interestingly, TiO_2_ nanoparticles have been shown to modulate the biological response of dendritic cells leading to increased levels of ROS (reactive oxygen species), TNF-a, IL -1β, and IL-6 [38,60,61]. Therefore, the findings of the current study align with other research findings indicating the potential of TiO_2_ nanoparticles to regulate biological processes.

*Concluding remarks: In vitro* models are known to provide some advantages on the development of new materials. For instance, in vitro testing offers a framework for testing and validating the mechanism of action of many different “ingredients” or products directly on the cellular and molecular levels, may serve as a reliable alternative for the use of animal models, and may speed up the understanding of the molecular mechanisms involved. Nevertheless, one must consider that limitations of in vitro methods include the fact that it does not fully represent the heterogeneity of biological tissues, and therefore, does not fully mimic its physiological and/or pathological responses. With that in mind, we are convinced that the findings of the present study postulate clear information to support the hypothesis that the presence of TiO_2_-nt in the composition of GIC has the potential to regulate biological responses and presents an attractive approach to improve the clinical outcomes of GICs.

## 6 Conclusion

TiO_2_-nt into GIC matrix was able to induce and reverse the inflammatory profile in NIH/3T3 cells by modulating pro- and anti-cytokines. This effect was observed not only in the absence of bacterial LPS but also when the system was challenged with LPS. Furthermore, it did not negatively impact the biological behavior of fibroblastic cells.

## Clinical significance statement

TiO_2_-nt into the GIC matrix was able to induce and reverse the inflammatory profile in NIH/3T3 cells by modulating pro- and anti-cytokines, not only in the absence of LPS but also when the system was challenged with LPS.

## Data availability statement

The data that support the findings of this study are available from the corresponding author upon reasonable request.

## CRediT authorship contribution statement

**João Pedro Rangel Coelho:** Writing – original draft, Methodology, Funding acquisition, Conceptualization. **Pedro Viel Gogolla:** Writing – original draft, Methodology, Funding acquisition, Conceptualization. **Maria Dovoli Meyer:** Writing – original draft, Methodology, Data curation. **Lucas Carvalho Simão:** Writing – original draft, Methodology. **Bruna Carolina Costa:** Writing – original draft, Methodology, Formal analysis. **Renato Correa Viana Casarin:** Writing – original draft, Methodology, Formal analysis. **Mauro Pedrine Santamaria:** Writing – original draft, Resources. **Lucas Novaes Teixeira:** Writing – review & editing, Software. **Daiane Cristina Peruzzo:** Writing – original draft, Formal analysis. **Paulo Noronha Lisboa-Filho:** Writing – review & editing, Validation, Methodology, Investigation, Data curation. **Francisco Humberto Nociti-Jr:** Writing – review & editing, Visualization, Resources, Investigation, Conceptualization. **Kamila Rosamilia Kantovitz:** Writing – review & editing, Visualization, Supervision, Software, Resources, Project administration, Funding acquisition, Conceptualization.

## Declaration of competing interest

The authors declare the following financial interests/personal relationships which may be considered as potential competing interests:Kamila Rosamilia Kantovitz reports financial support was provided by State of Sao Paulo Research Foundation. Pedro Viel Gogolla reports was provided by State of Sao Paulo Research Foundation. Joao Pedro Coelho Rangel reports financial support was provided by State of Sao Paulo Research Foundation. If there are other authors, they declare that they have no known competing financial interests or personal relationships that could have appeared to influence the work reported in this paper.
